# *catena*-Poly[[sodium-di-μ-β-d-glucose] chloride]

**DOI:** 10.1107/S1600536809039993

**Published:** 2009-10-07

**Authors:** K. C. Wong, Abdul Hamid, S. Baharuddin, Ching Kheng Quah, Hoong-Kun Fun

**Affiliations:** a School of Chemical Sciences, Universiti Sains Malaysia, 11800 USM, Penang, Malaysia; b School of Biological Sciences, Universiti Sains Malaysia, 11800 USM, Penang, Malaysia; c X-ray Crystallography Unit, School of Physics, Universiti Sains Malaysia, 11800 USM, Penang, Malaysia

## Abstract

The asymmetric unit of the title compound, {[Na(C_6_H_12_O_6_)_2_]Cl}_*n*_, contains six glucose mol­ecules, three Na^+^ ions and three Cl^−^ ions, *i.e.* three independent {[Na(C_6_H_12_O_6_)_2_]Cl} units. Each of these units forms polymeric chains along the *c* axis. Each Na^+^ ion is surrounded by six O atoms from four glucose mol­ecules, forming a distorted octa­hedral geometry. All glucose mol­ecules adopt chair conformations. The constituent units are linked into a three-dimensional framework by O—H⋯Cl and O—H⋯O hydrogen bonds, utilizing all the O—H groups.

## Related literature

For general background to *H. sagittifolia* and its use in folk medicine, see: Duke (1985 ▶); Burkill (1966 ▶); Sulaiman & Boyce (2005 ▶). For the crystal structure of the monohydrated analogue, see: Ferguson *et al.* (1991 ▶). For ring conformations, see: Cremer & Pople (1975 ▶). For the stability of the temperature controller used for the data collection, see: Cosier & Glazer (1986 ▶).
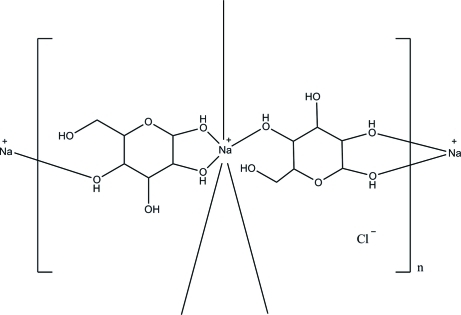


## Experimental

### 

#### Crystal data

[Na(C_6_H_12_O_6_)_2_]Cl
*M* *_r_* = 418.75
Trigonal, 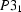

*a* = 16.3795 (4) Å
*c* = 17.4232 (6) Å
*V* = 4048.2 (2) Å^3^
*Z* = 9
Mo *K*α radiation
μ = 0.30 mm^−1^
*T* = 100 K
0.50 × 0.38 × 0.26 mm

#### Data collection

Bruker SMART APEXII CCD area-detector diffractometer
Absorption correction: multi-scan (**SADABS**; Bruker, 2005 ▶) *T*_min_ = 0.815, *T*_max_ = 0.928
211137 measured reflections
27007 independent reflections
25094 reflections with *I* > 2σ(*I*)
*R*_int_ = 0.050

#### Refinement

*R*[*F* ^2^ > 2σ(*F* ^2^)] = 0.042
*wR*(*F* ^2^) = 0.111
*S* = 1.06
27007 reflections
715 parameters
1 restraint
H-atom parameters constrained
Δρ_max_ = 0.80 e Å^−3^
Δρ_min_ = −0.40 e Å^−3^
Absolute structure: Flack (1983 ▶), 13472 Friedel pairs
Flack parameter: 0.02 (3)

### 

Data collection: *APEX2* (Bruker, 2005 ▶); cell refinement: *SAINT* (Bruker, 2005 ▶); data reduction: *SAINT*; program(s) used to solve structure: *SHELXTL* (Sheldrick, 2008 ▶); program(s) used to refine structure: *SHELXTL*; molecular graphics: *SHELXTL*; software used to prepare material for publication: *SHELXTL* and *PLATON* (Spek, 2009 ▶).

## Supplementary Material

Crystal structure: contains datablocks global, I. DOI: 10.1107/S1600536809039993/ci2906sup1.cif

Structure factors: contains datablocks I. DOI: 10.1107/S1600536809039993/ci2906Isup2.hkl

Additional supplementary materials:  crystallographic information; 3D view; checkCIF report

## Figures and Tables

**Table 1:** Hydrogen-bond geometry (Å, °)

*D*—H⋯*A*	*D*—H	H⋯*A*	*D*⋯*A*	*D*—H⋯*A*
O2*A*—H2*A*⋯O12*B*^i^	0.85	1.79	2.630 (3)	172
O2*B*—H2*B*⋯O12*C*^ii^	0.85	1.79	2.640 (3)	174
O2*C*—H2*C*⋯O6*A*^iii^	0.85	1.82	2.665 (3)	176
O3*A*—H3*A*⋯O1*B*	0.85	1.91	2.753 (2)	169
O3*B*—H3*B*⋯O1*C*^iv^	0.85	1.90	2.727 (2)	163
O3*C*—H3*C*⋯O7*A*^v^	0.85	1.94	2.755 (2)	161
O4*A*—H4*A*⋯Cl2^vi^	0.82	2.34	3.158 (2)	174
O4*B*—H4*B*⋯Cl3^vii^	0.82	2.45	3.270 (2)	173
O4*C*—H4*C*⋯Cl1	0.82	2.47	3.256 (2)	161
O5*A*—H5*A*⋯Cl2^viii^	0.83	2.35	3.151 (1)	162
O5*B*—H5*B*⋯O10*B*^iii^	0.85	1.98	2.828 (2)	175
O5*C*—H5*C*⋯O10*C*^ix^	0.85	1.94	2.773 (2)	165
O6*A*—H6*A*⋯Cl2^viii^	0.82	2.33	3.144 (2)	174
O6*B*—H6*B*⋯Cl3^x^	0.82	2.41	3.220 (2)	170
O6*C*—H6*C*⋯O8*B*^xi^	0.82	2.33	2.696 (3)	108
O8*A*—H8*A*⋯O6*B*	0.85	1.88	2.726 (3)	177
O8*B*—H8*B*⋯O6*C*^iv^	0.93	2.10	2.696 (3)	121
O8*C*—H8*C*⋯O12*A*^v^	0.85	1.85	2.699 (2)	173
O9*A*—H9*A*⋯O7*B*^i^	0.85	1.97	2.786 (3)	162
O9*B*—H9*B*⋯O7*C*^ii^	0.85	1.97	2.798 (2)	166
O9*C*—H9*C*⋯O1*A*^iii^	0.85	1.94	2.775 (2)	168
O10*A*—H10*A*⋯Cl2^xii^	0.82	2.51	3.245 (2)	150
O10*B*—H10*B*⋯Cl3^xiii^	0.82	2.33	3.147 (2)	171
O10*C*—H10*C*⋯Cl1^xi^	0.82	2.48	3.144 (2)	139
O11*A*—H11*A*⋯O4*A*^i^	0.85	2.03	2.819 (3)	155
O11*B*—H11*B*⋯Cl3^vi^	0.81	2.43	3.159 (2)	150
O11*C*—H11*C*⋯Cl1^xiv^	0.83	2.36	3.166 (2)	164
O12*A*—H12*A*⋯Cl2	0.82	2.45	3.229 (2)	159
O12*B*—H12*B*⋯Cl3^vi^	0.82	2.37	3.135 (2)	156
O12*C*—H12*C*⋯Cl1^xiv^	0.82	2.32	3.138 (2)	173

Symmetry codes: (i) 
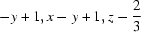
; (ii) 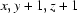; (iii) 
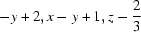
; (iv) 
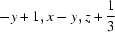
; (v) 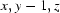; (vi) 
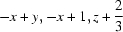
; (vii) 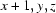; (viii) 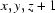; (ix) 
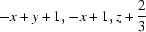
; (x) 
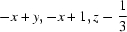
; (xi) 
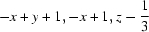
; (xii) 
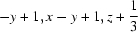
; (xiii) 
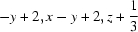
; (xiv) 
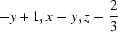
.

**Fig. 1.**
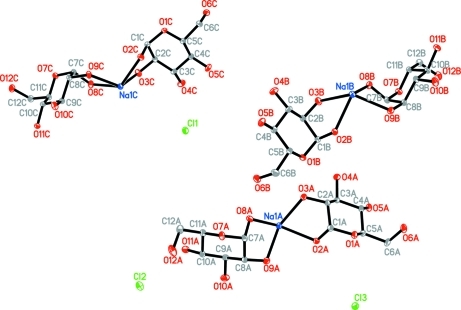
The asymmetric unit of the title compound, showing the atom-numbering scheme. H atoms have been omitted for clarity.

**Fig. 2.**
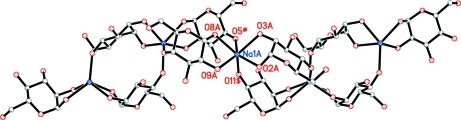
Part of a polymeric chain formed by Na1A ions, showing the coordination environment. Displacement ellipsoids are drawn at the 50% probability level. H atoms have been omitted for clarity. Symmetry codes: (#) -x+y, 1-x, -1/3+z; ($) 1-y, 1+x-y, 1/3+z.

**Fig. 3.**
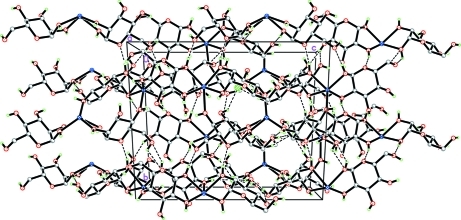
The crystal packing of the title compound, viewed down the a axis. Hydrogen bonds are shown as dashed lines. C-bound H atoms have been omitted for clarity.

## supplementary crystallographic information

### Comment

Research in phytochemical study often results in discovery of novel and interesting secondary metabolites from medical plants. *Homalomena sagittifolia* Jungh, which belongs to Araceae family, is a small herb found growing in damp, wet and low open spaces in the forest of Malaysia. The genus *Homalomena* has an overwhelmingly Asian distribution with greatest number of species occurring in archipelagic Malaysia as reported by Sulaiman & Boyce (2005). The folklores regarding the species are rich enough to be mentioned in CRC hand book of medicinal plants (Duke, 1985). In Malaysia, leaves of *H. sagittifolia* are used as a poultice to cure leg sores. A decoction of the rhizomes and leaves is given to drink for fever (Burkill, 1966). So far, no extensive phytochemical study of *H. sagittifolia* has been performed. As part of our on-going search for chemical constituents, we investigated the leaves of *H. sagittifolia*, which lead to isolation of the title compound. Herein, we report the crystal structure of the title compound.

The asymmetric unit contains three independent [Na(C_6_H_12_O_6_)_2_]^+^ units [say A, B and C] and three Cl^-^ ions (Fig. 1). Each of these independent units form polymeric chains along the *c*-axis. Each Na^+^ ion is surrounded by six O atoms from two independent and two symmetry related glucose molecules to form a distorted octahedral geometry, with Na—O distances ranging from 2.3433 (16) to 2.3966 (15) Å, 2.3455 (15) to 2.3957 (16) Å and 2.3040 (16) to 2.4372 (16) Å, for units *A*, *B* and *C*, respectively, whereas, the angles around the Na^+^ ion range from 68.64 (5) to 138.92 (6)°, 68.99 (5) to 138.99 (6)° and 69.07 (5) to 137.05 (6)° for units *A*, *B* and *C*, respectively. Bond lengths and angles are within normal ranges, and comparable to a closely related structure (Ferguson *et al.*, 1991). All the six independent glucose units in the asymmetric unit adopt chair conformations. The puckering parameters (Cremer & Pople, 1975) are Q = 0.580 (2) Å, Θ = 2.1 (2)° and φ = 333 (6)° for O1A/C1A–C5A ring, Q = 0.570 (2) Å, Θ = 1.3 (2)° and φ = 83 (12)° for O7A/C7A–C11A ring, Q = 0.573 (2) Å, Θ = 0.0 (2)° and φ = 31 (8)° for O1B/C1B–C5B) ring, Q = 0.577 (2) Å, Θ = 0.0 (2)° and φ = 9 (11)° for O7B/C7B–C11B ring, Q = 0.570 (2) Å, Θ = 0.0 (2)° and φ = 66 (6)° for O1C/C1C–C5C ring, and Q = 0.580 (2) Å; Θ = 0.4 (2)° and φ = 3 (10)° for O7C/C7C–C11C ring.

In the crystal, the constituent units are linked into a three-dimensional framework (Fig. 3) by O—H···Cl and O—H···O hydrogen bonds (Table 1), utilizing all the O-H groups.

### Experimental

The title compound was isolated from the methanolic extract of the leaves of *H. sagittifolia*. The crude extract was fractionated by using Sephadex LH-20 CC eluting with methanol-water (1:1). Fraction-9 was subjected to silica-gel column chromatography, repeatedly eluting with chloroform-methanol solvent mixture with their ratio ranging from 90:10–70:30. Finally, colourless single crystals were grown from a methanol-acetone solution.

### Refinement

O-bound H atoms were initially located in difference maps and later their positions were fixed, with O-H = 0.81–0.93 Å, and refined as riding with the parent atom with U_iso_(H) = 1.5U_eq_(O). C-bound H atoms were placed in calculated positions, with C–H = 0.97 and 0.98 Å, and refined using a riding model, with *U*_iso_(H) = 1.2 *U*_eq_(C). The crystal under investigation was twinned and the use of the twin law (1 1 0/0 -1 0/ 0 0 -1) resulted in a BASF parameter (SHELXTL; Sheldrick, 2008) of 0.3315 (8).

### Crystal data

**Table:**

[Na(C_6_H_12_O_6_)_2_]Cl	*D*_x_ = 1.546 Mg m^−^^3^
*M**_r_* = 418.75	Mo *K*α radiation, λ = 0.71073 Å
Trigonal, *P*3_1_	Cell parameters from 9874 reflections
Hall symbol: P 31	θ = 2.5–34.9°
*a* = 16.3795 (4) Å	µ = 0.30 mm^−^^1^
*c* = 17.4232 (6) Å	*T* = 100 K
*V* = 4048.2 (2) Å^3^	Block, colourless
*Z* = 9	0.50 × 0.38 × 0.26 mm
*F*(000) = 1980	

### Data collection

**Table:**

Bruker SMART APEXII CCD area-detector diffractometer	27007 independent reflections
Radiation source: fine-focus sealed tube	25094 reflections with *I* > 2σ(*I*)
graphite	*R*_int_ = 0.050
φ and ω scans	θ_max_ = 36.8°, θ_min_ = 1.2°
Absorption correction: multi-scan (*SADABS*; Bruker, 2005)	*h* = −27→27
*T*_min_ = 0.815, *T*_max_ = 0.928	*k* = −27→27
211137 measured reflections	*l* = −29→29

### Refinement

**Table:**

Refinement on *F*^2^	Secondary atom site location: difference Fourier map
Least-squares matrix: full	Hydrogen site location: inferred from neighbouring sites
*R*[*F*^2^ > 2σ(*F*^2^)] = 0.042	H-atom parameters constrained
*wR*(*F*^2^) = 0.111	*w* = 1/[σ^2^(*F*_o_^2^) + (0.0718*P*)^2^] where *P* = (*F*_o_^2^ + 2*F*_c_^2^)/3
*S* = 1.06	(Δ/σ)_max_ = 0.001
27007 reflections	Δρ_max_ = 0.80 e Å^−^^3^
715 parameters	Δρ_min_ = −0.40 e Å^−^^3^
1 restraint	Absolute structure: Flack (1983), 13472 Friedel pairs
Primary atom site location: structure-invariant direct methods	Flack parameter: 0.02 (3)

### Special details

**Table:**

Experimental. The crystal was placed in the cold stream of an Oxford Cyrosystems Cobra open-flow nitrogen cryostat (Cosier & Glazer, 1986) operating at 100.0 (1) K.
Geometry. All esds (except the esd in the dihedral angle between two l.s. planes) are estimated using the full covariance matrix. The cell esds are taken into account individually in the estimation of esds in distances, angles and torsion angles; correlations between esds in cell parameters are only used when they are defined by crystal symmetry. An approximate (isotropic) treatment of cell esds is used for estimating esds involving l.s. planes.
Refinement. Refinement of F^2^ against ALL reflections. The weighted R-factor wR and goodness of fit S are based on F^2^, conventional R-factors R are based on F, with F set to zero for negative F^2^. The threshold expression of F^2^ > 2sigma(F^2^) is used only for calculating R-factors(gt) etc. and is not relevant to the choice of reflections for refinement. R-factors based on F^2^ are statistically about twice as large as those based on F, and R- factors based on ALL data will be even larger.

### Fractional atomic coordinates and isotropic or equivalent isotropic displacement parameters (Å^2^)

**Table:**

	*x*	*y*	*z*	*U*_iso_*/*U*_eq_	
Na1A	0.40243 (6)	0.79506 (6)	0.70138 (4)	0.01379 (13)	
O1A	0.42335 (10)	0.97603 (9)	0.90774 (7)	0.0147 (2)	
O2A	0.37480 (10)	0.88583 (10)	0.79491 (8)	0.0161 (2)	
H2A	0.3487	0.9164	0.7792	0.024*	
O3A	0.51727 (9)	0.84858 (10)	0.80086 (8)	0.0155 (2)	
H3A	0.5753	0.8645	0.8013	0.023*	
O4A	0.47353 (11)	0.75675 (11)	0.95023 (8)	0.0184 (2)	
H4A	0.4628	0.7117	0.9229	0.028*	
O5A	0.32729 (10)	0.76721 (9)	1.03053 (7)	0.0155 (2)	
H5A	0.3395	0.7896	1.0745	0.023*	
O6A	0.39899 (12)	0.99572 (10)	1.07224 (8)	0.0208 (3)	
H6A	0.4010	0.9595	1.1037	0.031*	
O7A	0.56259 (10)	0.94644 (9)	0.48723 (8)	0.0152 (2)	
O8A	0.51828 (9)	0.86139 (10)	0.60264 (8)	0.0154 (2)	
H8A	0.5747	0.8829	0.6178	0.023*	
O9A	0.36105 (10)	0.87692 (11)	0.61387 (8)	0.0180 (2)	
H9A	0.3206	0.8949	0.6118	0.027*	
O10A	0.27167 (10)	0.77955 (11)	0.47331 (9)	0.0213 (3)	
H10A	0.2351	0.7350	0.5001	0.032*	
O11A	0.39410 (10)	0.74111 (10)	0.37729 (8)	0.0180 (2)	
H11A	0.3626	0.7434	0.3395	0.027*	
O12A	0.58050 (12)	0.95678 (11)	0.32001 (9)	0.0255 (3)	
H12A	0.5305	0.9282	0.2967	0.038*	
C1A	0.45104 (12)	0.94345 (12)	0.84180 (10)	0.0139 (3)	
H1D	0.4971	0.9979	0.8121	0.017*	
C2A	0.49595 (12)	0.88519 (12)	0.86730 (10)	0.0125 (3)	
H2D	0.5542	0.9257	0.8956	0.015*	
C3A	0.42781 (12)	0.80420 (12)	0.91894 (9)	0.0125 (2)	
H3D	0.3728	0.7596	0.8889	0.015*	
C4A	0.39598 (12)	0.84212 (12)	0.98499 (10)	0.0129 (3)	
H4D	0.4506	0.8823	1.0172	0.015*	
C5A	0.35402 (12)	0.90065 (12)	0.95469 (10)	0.0131 (3)	
H5D	0.2986	0.8604	0.9232	0.016*	
C6A	0.32521 (13)	0.94381 (13)	1.01897 (11)	0.0180 (3)	
H6D	0.3052	0.9853	0.9968	0.022*	
H6E	0.2718	0.8939	1.0461	0.022*	
C7A	0.51247 (12)	0.92956 (12)	0.55789 (10)	0.0138 (3)	
H7D	0.5390	0.9886	0.5869	0.017*	
C8A	0.40834 (12)	0.89450 (12)	0.54197 (10)	0.0137 (3)	
H8D	0.4026	0.9437	0.5147	0.016*	
C9A	0.36633 (12)	0.80596 (12)	0.49278 (10)	0.0143 (3)	
H9D	0.3667	0.7548	0.5217	0.017*	
C10A	0.42518 (12)	0.82541 (12)	0.41974 (10)	0.0139 (3)	
H10D	0.4188	0.8714	0.3881	0.017*	
C11A	0.52941 (12)	0.86400 (11)	0.43898 (10)	0.0139 (3)	
H11D	0.5357	0.8156	0.4671	0.017*	
C12A	0.59197 (14)	0.89208 (14)	0.36868 (11)	0.0199 (3)	
H12D	0.6573	0.9211	0.3848	0.024*	
H12E	0.5773	0.8359	0.3395	0.024*	
Na1B	0.87325 (6)	0.92592 (5)	1.03422 (4)	0.01344 (13)	
O1B	0.70647 (9)	0.91250 (10)	0.82118 (8)	0.0149 (2)	
O2B	0.79165 (10)	0.95978 (10)	0.93703 (8)	0.0159 (2)	
H2B	0.7640	0.9881	0.9544	0.024*	
O3B	0.79791 (11)	0.79928 (10)	0.94733 (8)	0.0183 (2)	
H3B	0.7838	0.7418	0.9462	0.028*	
O4B	0.89994 (11)	0.82099 (11)	0.80581 (9)	0.0207 (3)	
H4B	0.9544	0.8446	0.8213	0.031*	
O5B	0.91942 (9)	0.97609 (10)	0.71147 (8)	0.0171 (2)	
H5B	0.9321	0.9400	0.6854	0.026*	
O6B	0.69713 (11)	0.92403 (12)	0.65406 (9)	0.0221 (3)	
H6B	0.7308	0.9150	0.6245	0.033*	
O7B	0.70408 (9)	0.75684 (9)	1.24067 (7)	0.0139 (2)	
O8B	0.79839 (10)	0.80594 (9)	1.12998 (8)	0.0151 (2)	
H8B	0.8114	0.7573	1.1369	0.023*	
O9B	0.81132 (10)	0.97772 (10)	1.13269 (8)	0.0155 (2)	
H9B	0.7926	1.0176	1.1343	0.023*	
O10B	0.90017 (11)	1.03683 (10)	1.28298 (8)	0.0186 (2)	
H10B	0.9472	1.0735	1.2586	0.028*	
O11B	0.90851 (9)	0.89131 (10)	1.36548 (7)	0.0157 (2)	
H11B	0.8911	0.8745	1.4092	0.024*	
O12B	0.68312 (10)	0.71785 (11)	1.40569 (8)	0.0206 (3)	
H12B	0.7132	0.7455	1.4441	0.031*	
C1B	0.72851 (12)	0.88272 (13)	0.89236 (10)	0.0134 (3)	
H1E	0.6703	0.8446	0.9214	0.016*	
C2B	0.77495 (12)	0.82421 (12)	0.87540 (10)	0.0134 (3)	
H2E	0.7302	0.7668	0.8480	0.016*	
C3B	0.86262 (12)	0.88006 (12)	0.82614 (10)	0.0141 (3)	
H3E	0.9099	0.9341	0.8556	0.017*	
C4B	0.83738 (12)	0.91541 (12)	0.75406 (10)	0.0135 (3)	
H4E	0.7954	0.8613	0.7220	0.016*	
C5B	0.78801 (12)	0.97057 (12)	0.77426 (10)	0.0137 (3)	
H5E	0.8316	1.0268	0.8035	0.016*	
C6B	0.75485 (14)	1.00088 (14)	0.70404 (11)	0.0199 (3)	
H6F	0.7194	1.0306	0.7206	0.024*	
H6G	0.8094	1.0473	0.6758	0.024*	
C7B	0.73296 (12)	0.81654 (12)	1.17456 (9)	0.0125 (3)	
H7E	0.6775	0.8013	1.1432	0.015*	
C8B	0.77960 (12)	0.91972 (12)	1.19957 (9)	0.0124 (2)	
H8E	0.7333	0.9304	1.2267	0.015*	
C9B	0.86271 (12)	0.94398 (11)	1.25254 (9)	0.0127 (3)	
H9E	0.9120	0.9397	1.2240	0.015*	
C10B	0.82991 (11)	0.87510 (12)	1.31930 (9)	0.0118 (2)	
H10E	0.7854	0.8842	1.3507	0.014*	
C11B	0.78116 (12)	0.77340 (12)	1.29000 (9)	0.0126 (2)	
H11E	0.8267	0.7637	1.2607	0.015*	
C12B	0.74206 (13)	0.70209 (12)	1.35467 (10)	0.0167 (3)	
H12F	0.7060	0.6393	1.3330	0.020*	
H12G	0.7941	0.7049	1.3835	0.020*	
Na1C	0.72272 (5)	0.26194 (6)	0.36712 (4)	0.01372 (13)	
O1C	0.87438 (9)	0.25866 (10)	0.58123 (8)	0.0150 (2)	
O2C	0.83296 (9)	0.30390 (10)	0.46694 (8)	0.0158 (2)	
H2C	0.8882	0.3340	0.4483	0.024*	
O3C	0.67052 (10)	0.13599 (10)	0.45499 (8)	0.0180 (2)	
H3C	0.6325	0.0769	0.4548	0.027*	
O4C	0.58660 (10)	0.15191 (10)	0.59700 (9)	0.0205 (3)	
H4C	0.5568	0.1799	0.5959	0.031*	
O5C	0.71738 (10)	0.31138 (10)	0.68982 (9)	0.0183 (2)	
H5C	0.6798	0.2779	0.7252	0.027*	
O6C	0.89548 (12)	0.26778 (14)	0.74742 (9)	0.0251 (3)	
H6C	0.9069	0.2317	0.7231	0.038*	
O7C	0.73582 (10)	0.09477 (9)	0.16154 (7)	0.0149 (2)	
O8C	0.68727 (10)	0.13749 (9)	0.27402 (8)	0.0166 (2)	
H8C	0.6568	0.0796	0.2865	0.025*	
O9C	0.83698 (9)	0.31498 (10)	0.27036 (8)	0.0156 (2)	
H9C	0.8941	0.3590	0.2676	0.023*	
O10C	0.79948 (11)	0.36892 (9)	0.12121 (8)	0.0177 (2)	
H10C	0.8187	0.4069	0.1567	0.027*	
O11C	0.64855 (10)	0.21736 (10)	0.03950 (7)	0.0150 (2)	
H11C	0.6674	0.2224	−0.0056	0.022*	
O12C	0.71783 (12)	0.05674 (11)	−0.00376 (8)	0.0207 (3)	
H12C	0.7264	0.1011	−0.0311	0.031*	
C1C	0.82406 (12)	0.22769 (13)	0.51041 (10)	0.0143 (3)	
H1F	0.8479	0.1931	0.4808	0.017*	
C2C	0.71885 (12)	0.16317 (12)	0.52677 (10)	0.0135 (3)	
H2F	0.7096	0.1069	0.5541	0.016*	
C3C	0.68206 (12)	0.21485 (12)	0.57588 (10)	0.0138 (3)	
H3F	0.6855	0.2675	0.5465	0.017*	
C4C	0.74205 (12)	0.25268 (12)	0.64813 (10)	0.0140 (3)	
H4F	0.7322	0.1996	0.6806	0.017*	
C5C	0.84708 (12)	0.31259 (13)	0.62873 (10)	0.0143 (3)	
H5F	0.8573	0.3685	0.6002	0.017*	
C6C	0.91000 (14)	0.34435 (15)	0.69944 (11)	0.0193 (3)	
H6H	0.9754	0.3783	0.6833	0.023*	
H6I	0.8978	0.3874	0.7288	0.023*	
C7C	0.76415 (12)	0.15386 (12)	0.22817 (9)	0.0131 (3)	
H7F	0.8080	0.1427	0.2585	0.016*	
C8C	0.81344 (12)	0.25722 (12)	0.20314 (10)	0.0123 (3)	
H8F	0.8713	0.2725	0.1752	0.015*	
C9C	0.74880 (12)	0.27549 (12)	0.15133 (10)	0.0128 (3)	
H9F	0.6942	0.2674	0.1807	0.015*	
C10C	0.71611 (12)	0.20734 (12)	0.08441 (10)	0.0125 (3)	
H10F	0.7705	0.2207	0.0522	0.015*	
C11C	0.67005 (12)	0.10534 (12)	0.11347 (9)	0.0127 (3)	
H11F	0.6140	0.0912	0.1437	0.015*	
C12C	0.64163 (14)	0.03467 (13)	0.04824 (11)	0.0172 (3)	
H12H	0.6195	−0.0277	0.0695	0.021*	
H12I	0.5899	0.0333	0.0201	0.021*	
Cl1	0.47325 (3)	0.25978 (4)	0.54724 (3)	0.02033 (8)	
Cl2	0.42150 (4)	0.86126 (3)	0.18827 (3)	0.02115 (8)	
Cl3	0.12199 (4)	0.92863 (4)	0.85610 (3)	0.02089 (9)	

### Atomic displacement parameters (Å^2^)

**Table:**

	*U*^11^	*U*^22^	*U*^33^	*U*^12^	*U*^13^	*U*^23^
Na1A	0.0151 (3)	0.0151 (3)	0.0119 (3)	0.0081 (3)	0.0010 (2)	0.0004 (2)
O1A	0.0206 (6)	0.0113 (5)	0.0130 (5)	0.0085 (4)	0.0028 (4)	0.0017 (4)
O2A	0.0204 (6)	0.0188 (6)	0.0139 (5)	0.0135 (5)	−0.0035 (4)	−0.0023 (4)
O3A	0.0134 (5)	0.0211 (6)	0.0139 (5)	0.0100 (5)	0.0004 (4)	−0.0030 (4)
O4A	0.0273 (7)	0.0211 (6)	0.0149 (5)	0.0183 (6)	0.0009 (5)	0.0035 (4)
O5A	0.0191 (6)	0.0119 (5)	0.0123 (5)	0.0052 (4)	0.0033 (4)	0.0014 (4)
O6A	0.0338 (7)	0.0143 (6)	0.0149 (6)	0.0124 (5)	−0.0020 (5)	−0.0033 (4)
O7A	0.0170 (5)	0.0110 (5)	0.0124 (5)	0.0030 (4)	0.0012 (4)	−0.0010 (4)
O8A	0.0135 (5)	0.0186 (6)	0.0138 (5)	0.0078 (4)	−0.0006 (4)	0.0032 (4)
O9A	0.0206 (6)	0.0238 (6)	0.0154 (5)	0.0154 (5)	0.0042 (4)	0.0044 (5)
O10A	0.0147 (6)	0.0213 (6)	0.0286 (7)	0.0094 (5)	−0.0051 (5)	−0.0033 (5)
O11A	0.0192 (6)	0.0147 (5)	0.0202 (6)	0.0087 (5)	−0.0071 (5)	−0.0067 (4)
O12A	0.0297 (8)	0.0183 (6)	0.0161 (6)	0.0027 (5)	−0.0004 (5)	0.0035 (5)
C1A	0.0169 (7)	0.0124 (6)	0.0126 (7)	0.0074 (5)	0.0012 (5)	0.0007 (5)
C2A	0.0132 (6)	0.0134 (6)	0.0109 (6)	0.0066 (5)	0.0000 (5)	−0.0005 (5)
C3A	0.0154 (6)	0.0131 (6)	0.0107 (6)	0.0083 (5)	0.0003 (5)	−0.0003 (5)
C4A	0.0149 (6)	0.0114 (6)	0.0112 (6)	0.0057 (5)	0.0006 (5)	0.0002 (5)
C5A	0.0126 (6)	0.0125 (6)	0.0135 (6)	0.0058 (5)	0.0009 (5)	−0.0006 (5)
C6A	0.0225 (8)	0.0175 (7)	0.0168 (7)	0.0121 (6)	0.0047 (6)	−0.0011 (5)
C7A	0.0159 (6)	0.0129 (6)	0.0107 (6)	0.0058 (5)	0.0005 (5)	0.0006 (5)
C8A	0.0172 (7)	0.0137 (6)	0.0125 (6)	0.0093 (5)	−0.0003 (5)	0.0013 (5)
C9A	0.0131 (6)	0.0129 (6)	0.0162 (7)	0.0060 (5)	−0.0019 (5)	−0.0001 (5)
C10A	0.0167 (7)	0.0120 (6)	0.0133 (6)	0.0074 (5)	−0.0032 (5)	−0.0019 (5)
C11A	0.0159 (7)	0.0111 (6)	0.0128 (6)	0.0054 (5)	0.0009 (5)	0.0009 (5)
C12A	0.0196 (7)	0.0190 (7)	0.0176 (7)	0.0072 (6)	0.0037 (6)	0.0005 (6)
Na1B	0.0138 (3)	0.0139 (3)	0.0123 (3)	0.0067 (3)	−0.0006 (2)	0.0000 (2)
O1B	0.0129 (5)	0.0209 (6)	0.0122 (5)	0.0094 (5)	−0.0001 (4)	0.0019 (4)
O2B	0.0205 (6)	0.0171 (5)	0.0142 (5)	0.0123 (5)	−0.0038 (4)	−0.0047 (4)
O3B	0.0268 (7)	0.0152 (5)	0.0152 (6)	0.0121 (5)	−0.0044 (5)	−0.0018 (4)
O4B	0.0203 (6)	0.0194 (6)	0.0280 (7)	0.0141 (5)	0.0026 (5)	−0.0006 (5)
O5B	0.0142 (5)	0.0147 (5)	0.0200 (6)	0.0055 (4)	0.0061 (4)	0.0002 (4)
O6B	0.0169 (6)	0.0339 (8)	0.0159 (6)	0.0130 (6)	−0.0028 (5)	−0.0031 (5)
O7B	0.0103 (5)	0.0142 (5)	0.0135 (5)	0.0033 (4)	0.0000 (4)	0.0023 (4)
O8B	0.0181 (6)	0.0128 (5)	0.0140 (5)	0.0074 (4)	0.0028 (4)	0.0002 (4)
O9B	0.0217 (6)	0.0154 (5)	0.0137 (5)	0.0126 (5)	0.0031 (4)	0.0032 (4)
O10B	0.0225 (6)	0.0130 (5)	0.0157 (6)	0.0054 (5)	−0.0025 (5)	−0.0013 (4)
O11B	0.0122 (5)	0.0195 (6)	0.0126 (5)	0.0058 (4)	−0.0017 (4)	0.0020 (4)
O12B	0.0152 (6)	0.0228 (6)	0.0156 (5)	0.0033 (5)	0.0037 (4)	0.0013 (5)
C1B	0.0145 (6)	0.0165 (7)	0.0106 (6)	0.0088 (5)	−0.0007 (5)	−0.0007 (5)
C2B	0.0154 (6)	0.0127 (6)	0.0124 (6)	0.0073 (5)	−0.0010 (5)	−0.0016 (5)
C3B	0.0131 (6)	0.0130 (6)	0.0170 (7)	0.0071 (5)	0.0005 (5)	−0.0018 (5)
C4B	0.0119 (6)	0.0123 (6)	0.0140 (7)	0.0045 (5)	0.0017 (5)	−0.0009 (5)
C5B	0.0128 (6)	0.0160 (6)	0.0123 (6)	0.0072 (5)	−0.0005 (5)	0.0000 (5)
C6B	0.0185 (7)	0.0232 (8)	0.0190 (8)	0.0112 (7)	−0.0003 (6)	0.0035 (6)
C7B	0.0117 (6)	0.0129 (6)	0.0097 (6)	0.0036 (5)	−0.0001 (4)	0.0005 (5)
C8B	0.0145 (6)	0.0134 (6)	0.0104 (6)	0.0078 (5)	0.0001 (5)	0.0005 (5)
C9B	0.0131 (6)	0.0113 (6)	0.0117 (6)	0.0047 (5)	−0.0010 (5)	−0.0002 (5)
C10B	0.0110 (6)	0.0135 (6)	0.0100 (6)	0.0054 (5)	−0.0005 (4)	0.0013 (5)
C11B	0.0123 (6)	0.0138 (6)	0.0108 (6)	0.0058 (5)	0.0005 (5)	0.0011 (5)
C12B	0.0177 (7)	0.0146 (7)	0.0150 (6)	0.0059 (6)	0.0014 (5)	0.0037 (5)
Na1C	0.0135 (3)	0.0153 (3)	0.0120 (3)	0.0070 (3)	−0.0002 (2)	0.0004 (2)
O1C	0.0147 (5)	0.0220 (6)	0.0134 (5)	0.0130 (5)	−0.0006 (4)	−0.0016 (4)
O2C	0.0138 (5)	0.0167 (5)	0.0142 (5)	0.0055 (5)	0.0017 (4)	0.0049 (4)
O3C	0.0207 (6)	0.0137 (5)	0.0143 (5)	0.0046 (5)	−0.0028 (4)	0.0005 (4)
O4C	0.0137 (5)	0.0163 (6)	0.0288 (7)	0.0055 (5)	0.0053 (5)	0.0029 (5)
O5C	0.0190 (6)	0.0164 (6)	0.0213 (6)	0.0102 (5)	0.0057 (5)	−0.0022 (5)
O6C	0.0292 (7)	0.0432 (9)	0.0155 (6)	0.0274 (7)	0.0010 (5)	0.0039 (6)
O7C	0.0215 (6)	0.0151 (5)	0.0123 (5)	0.0122 (5)	−0.0030 (4)	−0.0031 (4)
O8C	0.0203 (6)	0.0143 (5)	0.0131 (5)	0.0070 (5)	0.0042 (4)	0.0020 (4)
O9C	0.0138 (5)	0.0160 (5)	0.0110 (5)	0.0029 (4)	−0.0009 (4)	−0.0031 (4)
O10C	0.0250 (6)	0.0110 (5)	0.0148 (6)	0.0071 (5)	−0.0006 (5)	0.0007 (4)
O11C	0.0181 (5)	0.0197 (5)	0.0126 (5)	0.0136 (5)	−0.0020 (4)	−0.0004 (4)
O12C	0.0317 (7)	0.0207 (6)	0.0163 (6)	0.0181 (6)	0.0026 (5)	−0.0015 (5)
C1C	0.0144 (6)	0.0176 (7)	0.0129 (6)	0.0094 (6)	−0.0002 (5)	0.0000 (5)
C2C	0.0139 (6)	0.0128 (6)	0.0132 (7)	0.0061 (5)	0.0008 (5)	0.0010 (5)
C3C	0.0113 (6)	0.0129 (6)	0.0175 (7)	0.0062 (5)	0.0027 (5)	0.0025 (5)
C4C	0.0155 (7)	0.0139 (6)	0.0149 (7)	0.0092 (6)	0.0024 (5)	0.0010 (5)
C5C	0.0153 (7)	0.0163 (7)	0.0138 (6)	0.0097 (6)	−0.0009 (5)	−0.0011 (5)
C6C	0.0187 (8)	0.0254 (8)	0.0164 (7)	0.0129 (7)	−0.0050 (6)	−0.0050 (6)
C7C	0.0168 (7)	0.0150 (6)	0.0100 (6)	0.0098 (6)	−0.0013 (5)	−0.0015 (5)
C8C	0.0129 (6)	0.0132 (6)	0.0108 (6)	0.0065 (5)	−0.0005 (5)	−0.0007 (5)
C9C	0.0148 (6)	0.0123 (6)	0.0125 (6)	0.0078 (5)	−0.0003 (5)	0.0001 (5)
C10C	0.0127 (6)	0.0145 (6)	0.0126 (6)	0.0085 (5)	−0.0009 (5)	−0.0003 (5)
C11C	0.0140 (6)	0.0128 (6)	0.0113 (6)	0.0066 (5)	−0.0011 (5)	−0.0010 (5)
C12C	0.0206 (7)	0.0157 (7)	0.0156 (7)	0.0093 (6)	−0.0016 (6)	−0.0029 (5)
Cl1	0.01720 (18)	0.0248 (2)	0.01575 (17)	0.00802 (16)	0.00034 (14)	−0.00187 (15)
Cl2	0.0249 (2)	0.01823 (18)	0.01642 (18)	0.00789 (16)	−0.00165 (15)	0.00035 (14)
Cl3	0.0260 (2)	0.0250 (2)	0.01662 (19)	0.01643 (18)	0.00284 (15)	0.00257 (15)

### Geometric parameters (Å, °)

**Table:**

Na1A—O9A	2.3433 (16)	O11B—Na1B^iii^	2.3895 (15)
Na1A—O11A^i^	2.3447 (16)	O11B—H11B	0.81
Na1A—O5A^ii^	2.3726 (15)	O12B—C12B	1.428 (2)
Na1A—O3A	2.3795 (15)	O12B—H12B	0.82
Na1A—O8A	2.3831 (15)	C1B—C2B	1.521 (2)
Na1A—O2A	2.3966 (15)	C1B—H1E	0.98
O1A—C1A	1.432 (2)	C2B—C3B	1.524 (2)
O1A—C5A	1.443 (2)	C2B—H2E	0.98
O2A—C1A	1.392 (2)	C3B—C4B	1.524 (3)
O2A—H2A	0.85	C3B—H3E	0.98
O3A—C2A	1.425 (2)	C4B—C5B	1.525 (2)
O3A—H3A	0.85	C4B—H4E	0.98
O4A—C3A	1.430 (2)	C5B—C6B	1.519 (3)
O4A—H4A	0.82	C5B—H5E	0.98
O5A—C4A	1.421 (2)	C6B—H6F	0.97
O5A—Na1A^i^	2.3726 (15)	C6B—H6G	0.97
O5A—H5A	0.83	C7B—C8B	1.529 (2)
O6A—C6A	1.420 (2)	C7B—H7E	0.98
O6A—H6A	0.82	C8B—C9B	1.524 (2)
O7A—C7A	1.428 (2)	C8B—H8E	0.98
O7A—C11A	1.446 (2)	C9B—C10B	1.519 (2)
O8A—C7A	1.404 (2)	C9B—H9E	0.98
O8A—H8A	0.85	C10B—C11B	1.531 (2)
O9A—C8A	1.424 (2)	C10B—H10E	0.98
O9A—H9A	0.85	C11B—C12B	1.515 (2)
O10A—C9A	1.427 (2)	C11B—H11E	0.98
O10A—H10A	0.82	C12B—H12F	0.97
O11A—C10A	1.418 (2)	C12B—H12G	0.97
O11A—Na1A^ii^	2.3447 (15)	Na1C—O5C^v^	2.3040 (16)
O11A—H11A	0.85	Na1C—O9C	2.3397 (15)
O12A—C12A	1.441 (3)	Na1C—O2C	2.3488 (15)
O12A—H12A	0.82	Na1C—O3C	2.3594 (16)
C1A—C2A	1.533 (2)	Na1C—O11C^vi^	2.4367 (15)
C1A—H1D	0.98	Na1C—O8C	2.4372 (16)
C2A—C3A	1.528 (2)	O1C—C1C	1.429 (2)
C2A—H2D	0.98	O1C—C5C	1.435 (2)
C3A—C4A	1.519 (2)	O2C—C1C	1.404 (2)
C3A—H3D	0.98	O2C—H2C	0.85
C4A—C5A	1.526 (2)	O3C—C2C	1.427 (2)
C4A—H4D	0.98	O3C—H3C	0.85
C5A—C6A	1.520 (2)	O4C—C3C	1.425 (2)
C5A—H5D	0.98	O4C—H4C	0.82
C6A—H6D	0.97	O5C—C4C	1.416 (2)
C6A—H6E	0.97	O5C—Na1C^vi^	2.3040 (16)
C7A—C8A	1.529 (2)	O5C—H5C	0.85
C7A—H7D	0.98	O6C—C6C	1.425 (3)
C8A—C9A	1.521 (2)	O6C—H6C	0.82
C8A—H8D	0.98	O7C—C7C	1.432 (2)
C9A—C10A	1.531 (3)	O7C—C11C	1.442 (2)
C9A—H9D	0.98	O8C—C7C	1.399 (2)
C10A—C11A	1.532 (2)	O8C—H8C	0.85
C10A—H10D	0.98	O9C—C8C	1.432 (2)
C11A—C12A	1.513 (2)	O9C—H9C	0.85
C11A—H11D	0.98	O10C—C9C	1.427 (2)
C12A—H12D	0.97	O10C—H10C	0.82
C12A—H12E	0.97	O11C—C10C	1.430 (2)
Na1B—O5B^iii^	2.3455 (15)	O11C—Na1C^v^	2.4367 (15)
Na1B—O9B	2.3565 (16)	O11C—H11C	0.8325
Na1B—O3B	2.3576 (16)	O12C—C12C	1.435 (2)
Na1B—O2B	2.3881 (16)	O12C—H12C	0.82
Na1B—O11B^iv^	2.3895 (15)	C1C—C2C	1.532 (2)
Na1B—O8B	2.3957 (16)	C1C—H1F	0.98
O1B—C1B	1.443 (2)	C2C—C3C	1.524 (2)
O1B—C5B	1.444 (2)	C2C—H2F	0.98
O2B—C1B	1.401 (2)	C3C—C4C	1.525 (3)
O2B—H2B	0.85	C3C—H3F	0.98
O3B—C2B	1.426 (2)	C4C—C5C	1.533 (3)
O3B—H3B	0.85	C4C—H4F	0.98
O4B—C3B	1.424 (2)	C5C—C6C	1.521 (2)
O4B—H4B	0.82	C5C—H5F	0.98
O5B—C4B	1.417 (2)	C6C—H6H	0.97
O5B—Na1B^iv^	2.3455 (15)	C6C—H6I	0.97
O5B—H5B	0.85	C7C—C8C	1.530 (2)
O6B—C6B	1.430 (3)	C7C—H7F	0.98
O6B—H6B	0.82	C8C—C9C	1.530 (2)
O7B—C7B	1.430 (2)	C8C—H8F	0.98
O7B—C11B	1.437 (2)	C9C—C10C	1.515 (2)
O8B—C7B	1.403 (2)	C9C—H9F	0.98
O8B—H8B	0.93	C10C—C11C	1.535 (2)
O9B—C8B	1.427 (2)	C10C—H10F	0.98
O9B—H9B	0.85	C11C—C12C	1.520 (2)
O10B—C9B	1.427 (2)	C11C—H11F	0.98
O10B—H10B	0.82	C12C—H12H	0.97
O11B—C10B	1.426 (2)	C12C—H12I	0.97
			
O9A—Na1A—O11A^i^	100.32 (6)	C4B—C3B—H3E	109.3
O9A—Na1A—O5A^ii^	137.26 (6)	O5B—C4B—C3B	110.84 (14)
O11A^i^—Na1A—O5A^ii^	73.65 (5)	O5B—C4B—C5B	107.65 (14)
O9A—Na1A—O3A	131.66 (6)	C3B—C4B—C5B	111.08 (14)
O11A^i^—Na1A—O3A	122.09 (6)	O5B—C4B—H4E	109.1
O5A^ii^—Na1A—O3A	80.95 (5)	C3B—C4B—H4E	109.1
O9A—Na1A—O8A	70.22 (5)	C5B—C4B—H4E	109.1
O11A^i^—Na1A—O8A	132.59 (6)	O1B—C5B—C6B	107.88 (14)
O5A^ii^—Na1A—O8A	83.08 (5)	O1B—C5B—C4B	109.28 (14)
O3A—Na1A—O8A	93.17 (5)	C6B—C5B—C4B	112.94 (15)
O9A—Na1A—O2A	83.63 (5)	O1B—C5B—H5E	108.9
O11A^i^—Na1A—O2A	99.34 (6)	C6B—C5B—H5E	108.9
O5A^ii^—Na1A—O2A	138.92 (6)	C4B—C5B—H5E	108.9
O3A—Na1A—O2A	68.64 (5)	O6B—C6B—C5B	112.93 (16)
O8A—Na1A—O2A	124.22 (6)	O6B—C6B—H6F	109.0
C1A—O1A—C5A	113.35 (13)	C5B—C6B—H6F	109.0
C1A—O2A—Na1A	115.85 (11)	O6B—C6B—H6G	109.0
C1A—O2A—H2A	109.6	C5B—C6B—H6G	109.0
Na1A—O2A—H2A	116.9	H6F—C6B—H6G	107.8
C2A—O3A—Na1A	115.58 (10)	O8B—C7B—O7B	111.93 (14)
C2A—O3A—H3A	109.5	O8B—C7B—C8B	107.71 (13)
Na1A—O3A—H3A	132.6	O7B—C7B—C8B	109.74 (13)
C3A—O4A—H4A	109.5	O8B—C7B—H7E	109.1
C4A—O5A—Na1A^i^	146.82 (11)	O7B—C7B—H7E	109.1
C4A—O5A—H5A	102.8	C8B—C7B—H7E	109.1
Na1A^i^—O5A—H5A	110.1	O9B—C8B—C9B	109.90 (14)
C6A—O6A—H6A	109.5	O9B—C8B—C7B	108.50 (13)
C7A—O7A—C11A	114.15 (13)	C9B—C8B—C7B	110.38 (14)
C7A—O8A—Na1A	114.11 (10)	O9B—C8B—H8E	109.3
C7A—O8A—H8A	109.6	C9B—C8B—H8E	109.3
Na1A—O8A—H8A	114.7	C7B—C8B—H8E	109.3
C8A—O9A—Na1A	113.84 (10)	O10B—C9B—C10B	108.10 (14)
C8A—O9A—H9A	109.4	O10B—C9B—C8B	110.79 (14)
Na1A—O9A—H9A	136.4	C10B—C9B—C8B	109.38 (14)
C9A—O10A—H10A	109.5	O10B—C9B—H9E	109.5
C10A—O11A—Na1A^ii^	146.21 (11)	C10B—C9B—H9E	109.5
C10A—O11A—H11A	107.0	C8B—C9B—H9E	109.5
Na1A^ii^—O11A—H11A	106.8	O11B—C10B—C9B	110.03 (13)
C12A—O12A—H12A	109.5	O11B—C10B—C11B	108.78 (13)
O2A—C1A—O1A	112.30 (14)	C9B—C10B—C11B	110.56 (14)
O2A—C1A—C2A	107.14 (14)	O11B—C10B—H10E	109.1
O1A—C1A—C2A	109.83 (14)	C9B—C10B—H10E	109.1
O2A—C1A—H1D	109.2	C11B—C10B—H10E	109.1
O1A—C1A—H1D	109.2	O7B—C11B—C12B	107.85 (14)
C2A—C1A—H1D	109.2	O7B—C11B—C10B	109.63 (13)
O3A—C2A—C3A	109.83 (14)	C12B—C11B—C10B	112.35 (14)
O3A—C2A—C1A	108.74 (13)	O7B—C11B—H11E	109.0
C3A—C2A—C1A	109.97 (14)	C12B—C11B—H11E	109.0
O3A—C2A—H2D	109.4	C10B—C11B—H11E	108.9
C3A—C2A—H2D	109.4	O12B—C12B—C11B	112.78 (15)
C1A—C2A—H2D	109.4	O12B—C12B—H12F	109.0
O4A—C3A—C4A	108.28 (13)	C11B—C12B—H12F	109.0
O4A—C3A—C2A	110.05 (14)	O12B—C12B—H12G	109.0
C4A—C3A—C2A	110.02 (13)	C11B—C12B—H12G	109.0
O4A—C3A—H3D	109.5	H12F—C12B—H12G	107.8
C4A—C3A—H3D	109.5	O5C^v^—Na1C—O9C	120.55 (6)
C2A—C3A—H3D	109.5	O5C^v^—Na1C—O2C	129.99 (6)
O5A—C4A—C3A	110.83 (13)	O9C—Na1C—O2C	93.99 (5)
O5A—C4A—C5A	108.95 (14)	O5C^v^—Na1C—O3C	102.86 (6)
C3A—C4A—C5A	110.45 (13)	O9C—Na1C—O3C	131.69 (7)
O5A—C4A—H4D	108.9	O2C—Na1C—O3C	70.42 (5)
C3A—C4A—H4D	108.9	O5C^v^—Na1C—O11C^vi^	73.36 (5)
C5A—C4A—H4D	108.9	O9C—Na1C—O11C^vi^	79.31 (5)
O1A—C5A—C6A	108.33 (14)	O2C—Na1C—O11C^vi^	79.83 (5)
O1A—C5A—C4A	108.95 (13)	O3C—Na1C—O11C^vi^	137.05 (6)
C6A—C5A—C4A	112.29 (14)	O5C^v^—Na1C—O8C	102.51 (6)
O1A—C5A—H5D	109.0	O9C—Na1C—O8C	69.07 (5)
C6A—C5A—H5D	109.1	O2C—Na1C—O8C	124.36 (6)
C4A—C5A—H5D	109.1	O3C—Na1C—O8C	82.53 (5)
O6A—C6A—C5A	113.01 (15)	O11C^vi^—Na1C—O8C	140.42 (6)
O6A—C6A—H6D	109.0	C1C—O1C—C5C	114.49 (13)
C5A—C6A—H6D	109.0	C1C—O2C—Na1C	114.60 (11)
O6A—C6A—H6E	109.0	C1C—O2C—H2C	109.3
C5A—C6A—H6E	109.0	Na1C—O2C—H2C	109.0
H6D—C6A—H6E	107.8	C2C—O3C—Na1C	113.20 (10)
O8A—C7A—O7A	111.84 (14)	C2C—O3C—H3C	109.6
O8A—C7A—C8A	107.98 (14)	Na1C—O3C—H3C	137.0
O7A—C7A—C8A	109.99 (14)	C3C—O4C—H4C	109.5
O8A—C7A—H7D	109.0	C4C—O5C—Na1C^vi^	145.32 (12)
O7A—C7A—H7D	109.0	C4C—O5C—H5C	107.1
C8A—C7A—H7D	109.0	Na1C^vi^—O5C—H5C	107.5
O9A—C8A—C9A	111.21 (15)	C6C—O6C—H6C	109.5
O9A—C8A—C7A	107.96 (14)	C7C—O7C—C11C	113.51 (13)
C9A—C8A—C7A	110.37 (14)	C7C—O8C—Na1C	113.85 (10)
O9A—C8A—H8D	109.1	C7C—O8C—H8C	109.3
C9A—C8A—H8D	109.1	Na1C—O8C—H8C	121.6
C7A—C8A—H8D	109.1	C8C—O9C—Na1C	116.28 (10)
O10A—C9A—C8A	109.15 (14)	C8C—O9C—H9C	109.4
O10A—C9A—C10A	109.95 (15)	Na1C—O9C—H9C	133.9
C8A—C9A—C10A	109.42 (14)	C9C—O10C—H10C	109.5
O10A—C9A—H9D	109.4	C10C—O11C—Na1C^v^	146.64 (11)
C8A—C9A—H9D	109.4	C10C—O11C—H11C	104.9
C10A—C9A—H9D	109.4	Na1C^v^—O11C—H11C	103.5
O11A—C10A—C9A	110.42 (14)	C12C—O12C—H12C	109.5
O11A—C10A—C11A	107.41 (14)	O2C—C1C—O1C	111.42 (15)
C9A—C10A—C11A	111.10 (14)	O2C—C1C—C2C	107.13 (14)
O11A—C10A—H10D	109.3	O1C—C1C—C2C	109.55 (14)
C9A—C10A—H10D	109.3	O2C—C1C—H1F	109.6
C11A—C10A—H10D	109.3	O1C—C1C—H1F	109.6
O7A—C11A—C12A	107.79 (14)	C2C—C1C—H1F	109.6
O7A—C11A—C10A	109.47 (13)	O3C—C2C—C3C	110.71 (15)
C12A—C11A—C10A	113.19 (14)	O3C—C2C—C1C	108.03 (14)
O7A—C11A—H11D	108.8	C3C—C2C—C1C	110.01 (14)
C12A—C11A—H11D	108.8	O3C—C2C—H2F	109.4
C10A—C11A—H11D	108.8	C3C—C2C—H2F	109.4
O12A—C12A—C11A	112.48 (16)	C1C—C2C—H2F	109.4
O12A—C12A—H12D	109.1	O4C—C3C—C2C	109.80 (14)
C11A—C12A—H12D	109.1	O4C—C3C—C4C	109.38 (15)
O12A—C12A—H12E	109.1	C2C—C3C—C4C	109.62 (14)
C11A—C12A—H12E	109.1	O4C—C3C—H3F	109.3
H12D—C12A—H12E	107.8	C2C—C3C—H3F	109.3
O5B^iii^—Na1B—O9B	121.01 (6)	C4C—C3C—H3F	109.3
O5B^iii^—Na1B—O3B	102.33 (6)	O5C—C4C—C3C	110.78 (14)
O9B—Na1B—O3B	130.86 (6)	O5C—C4C—C5C	106.77 (14)
O5B^iii^—Na1B—O2B	134.78 (6)	C3C—C4C—C5C	111.54 (14)
O9B—Na1B—O2B	92.35 (5)	O5C—C4C—H4F	109.2
O3B—Na1B—O2B	68.99 (5)	C3C—C4C—H4F	109.2
O5B^iii^—Na1B—O11B^iv^	73.49 (5)	C5C—C4C—H4F	109.2
O9B—Na1B—O11B^iv^	79.43 (5)	O1C—C5C—C6C	108.13 (14)
O3B—Na1B—O11B^iv^	138.99 (6)	O1C—C5C—C4C	109.18 (14)
O2B—Na1B—O11B^iv^	85.16 (6)	C6C—C5C—C4C	113.07 (15)
O5B^iii^—Na1B—O8B	96.95 (5)	O1C—C5C—H5F	108.8
O9B—Na1B—O8B	69.66 (5)	C6C—C5C—H5F	108.8
O3B—Na1B—O8B	84.11 (6)	C4C—C5C—H5F	108.8
O2B—Na1B—O8B	124.61 (6)	O6C—C6C—C5C	112.82 (17)
O11B^iv^—Na1B—O8B	136.67 (6)	O6C—C6C—H6H	109.0
C1B—O1B—C5B	113.80 (13)	C5C—C6C—H6H	109.0
C1B—O2B—Na1B	115.56 (11)	O6C—C6C—H6I	109.0
C1B—O2B—H2B	109.6	C5C—C6C—H6I	109.0
Na1B—O2B—H2B	112.8	H6H—C6C—H6I	107.8
C2B—O3B—Na1B	114.17 (11)	O8C—C7C—O7C	112.14 (14)
C2B—O3B—H3B	109.4	O8C—C7C—C8C	107.53 (13)
Na1B—O3B—H3B	136.2	O7C—C7C—C8C	109.27 (13)
C3B—O4B—H4B	109.5	O8C—C7C—H7F	109.3
C4B—O5B—Na1B^iv^	146.74 (11)	O7C—C7C—H7F	109.3
C4B—O5B—H5B	105.5	C8C—C7C—H7F	109.3
Na1B^iv^—O5B—H5B	105.5	O9C—C8C—C7C	108.46 (13)
C6B—O6B—H6B	109.5	O9C—C8C—C9C	109.71 (14)
C7B—O7B—C11B	113.19 (12)	C7C—C8C—C9C	110.53 (13)
C7B—O8B—Na1B	113.17 (10)	O9C—C8C—H8F	109.4
C7B—O8B—H8B	123.4	C7C—C8C—H8F	109.4
Na1B—O8B—H8B	123.4	C9C—C8C—H8F	109.4
C8B—O9B—Na1B	115.71 (10)	O10C—C9C—C10C	108.00 (14)
C8B—O9B—H9B	109.6	O10C—C9C—C8C	109.77 (14)
Na1B—O9B—H9B	133.3	C10C—C9C—C8C	109.51 (13)
C9B—O10B—H10B	109.5	O10C—C9C—H9F	109.8
C10B—O11B—Na1B^iii^	145.66 (11)	C10C—C9C—H9F	109.8
C10B—O11B—H11B	109.6	C8C—C9C—H9F	109.8
Na1B^iii^—O11B—H11B	104.7	O11C—C10C—C9C	110.19 (13)
C12B—O12B—H12B	109.5	O11C—C10C—C11C	108.54 (14)
O2B—C1B—O1B	111.69 (15)	C9C—C10C—C11C	110.39 (14)
O2B—C1B—C2B	107.40 (14)	O11C—C10C—H10F	109.2
O1B—C1B—C2B	109.54 (14)	C9C—C10C—H10F	109.2
O2B—C1B—H1E	109.4	C11C—C10C—H10F	109.2
O1B—C1B—H1E	109.4	O7C—C11C—C12C	107.80 (14)
C2B—C1B—H1E	109.4	O7C—C11C—C10C	109.40 (13)
O3B—C2B—C1B	107.24 (14)	C12C—C11C—C10C	112.27 (14)
O3B—C2B—C3B	111.27 (15)	O7C—C11C—H11F	109.1
C1B—C2B—C3B	110.44 (14)	C12C—C11C—H11F	109.1
O3B—C2B—H2E	109.3	C10C—C11C—H11F	109.1
C1B—C2B—H2E	109.3	O12C—C12C—C11C	112.57 (15)
C3B—C2B—H2E	109.3	O12C—C12C—H12H	109.1
O4B—C3B—C2B	109.15 (14)	C11C—C12C—H12H	109.1
O4B—C3B—C4B	110.08 (15)	O12C—C12C—H12I	109.1
C2B—C3B—C4B	109.82 (14)	C11C—C12C—H12I	109.1
O4B—C3B—H3E	109.3	H12H—C12C—H12I	107.8
C2B—C3B—H3E	109.3		
			
O9A—Na1A—O2A—C1A	−120.96 (12)	O4B—C3B—C4B—C5B	174.19 (14)
O11A^i^—Na1A—O2A—C1A	139.58 (12)	C2B—C3B—C4B—C5B	53.99 (18)
O5A^ii^—Na1A—O2A—C1A	63.79 (15)	C1B—O1B—C5B—C6B	−176.84 (15)
O3A—Na1A—O2A—C1A	18.66 (11)	C1B—O1B—C5B—C4B	60.01 (18)
O8A—Na1A—O2A—C1A	−60.15 (13)	O5B—C4B—C5B—O1B	−177.07 (13)
O9A—Na1A—O3A—C2A	72.64 (14)	C3B—C4B—C5B—O1B	−55.55 (18)
O11A^i^—Na1A—O3A—C2A	−74.62 (13)	O5B—C4B—C5B—C6B	62.84 (19)
O5A^ii^—Na1A—O3A—C2A	−138.74 (12)	C3B—C4B—C5B—C6B	−175.64 (15)
O8A—Na1A—O3A—C2A	138.79 (12)	O1B—C5B—C6B—O6B	−67.08 (19)
O2A—Na1A—O3A—C2A	13.12 (11)	C4B—C5B—C6B—O6B	53.8 (2)
O9A—Na1A—O8A—C7A	14.24 (11)	Na1B—O8B—C7B—O7B	−167.86 (10)
O11A^i^—Na1A—O8A—C7A	100.11 (13)	Na1B—O8B—C7B—C8B	−47.14 (15)
O5A^ii^—Na1A—O8A—C7A	160.32 (12)	C11B—O7B—C7B—O8B	58.45 (18)
O3A—Na1A—O8A—C7A	−119.20 (12)	C11B—O7B—C7B—C8B	−61.09 (18)
O2A—Na1A—O8A—C7A	−52.99 (13)	Na1B—O9B—C8B—C9B	83.07 (14)
O11A^i^—Na1A—O9A—C8A	−113.77 (12)	Na1B—O9B—C8B—C7B	−37.71 (16)
O5A^ii^—Na1A—O9A—C8A	−36.75 (16)	O8B—C7B—C8B—O9B	55.43 (17)
O3A—Na1A—O9A—C8A	93.99 (13)	O7B—C7B—C8B—O9B	177.51 (13)
O8A—Na1A—O9A—C8A	17.96 (12)	O8B—C7B—C8B—C9B	−65.06 (17)
O2A—Na1A—O9A—C8A	147.85 (12)	O7B—C7B—C8B—C9B	57.03 (18)
Na1A—O2A—C1A—O1A	−164.95 (10)	O9B—C8B—C9B—O10B	66.81 (17)
Na1A—O2A—C1A—C2A	−44.28 (16)	C7B—C8B—C9B—O10B	−173.55 (13)
C5A—O1A—C1A—O2A	57.76 (19)	O9B—C8B—C9B—C10B	−174.12 (13)
C5A—O1A—C1A—C2A	−61.35 (18)	C7B—C8B—C9B—C10B	−54.48 (18)
Na1A—O3A—C2A—C3A	80.70 (14)	Na1B^iii^—O11B—C10B—C9B	−27.9 (3)
Na1A—O3A—C2A—C1A	−39.69 (16)	Na1B^iii^—O11B—C10B—C11B	93.4 (2)
O2A—C1A—C2A—O3A	54.06 (18)	O10B—C9B—C10B—O11B	−64.70 (18)
O1A—C1A—C2A—O3A	176.29 (13)	C8B—C9B—C10B—O11B	174.57 (13)
O2A—C1A—C2A—C3A	−66.23 (17)	O10B—C9B—C10B—C11B	175.12 (13)
O1A—C1A—C2A—C3A	55.99 (18)	C8B—C9B—C10B—C11B	54.40 (18)
O3A—C2A—C3A—O4A	67.34 (17)	C7B—O7B—C11B—C12B	−176.56 (14)
C1A—C2A—C3A—O4A	−173.02 (13)	C7B—O7B—C11B—C10B	60.83 (17)
O3A—C2A—C3A—C4A	−173.43 (13)	O11B—C10B—C11B—O7B	−177.59 (12)
C1A—C2A—C3A—C4A	−53.80 (18)	C9B—C10B—C11B—O7B	−56.66 (17)
Na1A^i^—O5A—C4A—C3A	−27.8 (3)	O11B—C10B—C11B—C12B	62.51 (18)
Na1A^i^—O5A—C4A—C5A	93.9 (2)	C9B—C10B—C11B—C12B	−176.56 (14)
O4A—C3A—C4A—O5A	−63.94 (18)	O7B—C11B—C12B—O12B	−68.48 (18)
C2A—C3A—C4A—O5A	175.76 (14)	C10B—C11B—C12B—O12B	52.5 (2)
O4A—C3A—C4A—C5A	175.23 (14)	O5C^v^—Na1C—O2C—C1C	107.60 (13)
C2A—C3A—C4A—C5A	54.93 (18)	O9C—Na1C—O2C—C1C	−116.03 (12)
C1A—O1A—C5A—C6A	−175.84 (14)	O3C—Na1C—O2C—C1C	17.03 (11)
C1A—O1A—C5A—C4A	61.74 (18)	O11C^vi^—Na1C—O2C—C1C	165.59 (12)
O5A—C4A—C5A—O1A	−179.34 (13)	O8C—Na1C—O2C—C1C	−48.74 (13)
C3A—C4A—C5A—O1A	−57.39 (17)	O5C^v^—Na1C—O3C—C2C	−112.48 (12)
O5A—C4A—C5A—C6A	60.66 (18)	O9C—Na1C—O3C—C2C	93.14 (13)
C3A—C4A—C5A—C6A	−177.39 (14)	O2C—Na1C—O3C—C2C	15.72 (11)
O1A—C5A—C6A—O6A	−67.48 (19)	O11C^vi^—Na1C—O3C—C2C	−33.19 (16)
C4A—C5A—C6A—O6A	52.9 (2)	O8C—Na1C—O3C—C2C	146.33 (12)
Na1A—O8A—C7A—O7A	−162.57 (10)	O5C^v^—Na1C—O8C—C7C	135.81 (11)
Na1A—O8A—C7A—C8A	−41.42 (16)	O9C—Na1C—O8C—C7C	17.61 (11)
C11A—O7A—C7A—O8A	59.60 (19)	O2C—Na1C—O8C—C7C	−62.54 (13)
C11A—O7A—C7A—C8A	−60.38 (18)	O3C—Na1C—O8C—C7C	−122.60 (12)
Na1A—O9A—C8A—C9A	76.24 (15)	O11C^vi^—Na1C—O8C—C7C	56.88 (15)
Na1A—O9A—C8A—C7A	−44.98 (16)	O5C^v^—Na1C—O9C—C8C	−78.21 (13)
O8A—C7A—C8A—O9A	56.58 (18)	O2C—Na1C—O9C—C8C	139.66 (12)
O7A—C7A—C8A—O9A	178.88 (13)	O3C—Na1C—O9C—C8C	72.47 (14)
O8A—C7A—C8A—C9A	−65.16 (18)	O11C^vi^—Na1C—O9C—C8C	−141.48 (12)
O7A—C7A—C8A—C9A	57.14 (18)	O8C—Na1C—O9C—C8C	14.28 (11)
O9A—C8A—C9A—O10A	65.19 (18)	Na1C—O2C—C1C—O1C	−164.10 (10)
C7A—C8A—C9A—O10A	−175.01 (14)	Na1C—O2C—C1C—C2C	−44.29 (16)
O9A—C8A—C9A—C10A	−174.45 (13)	C5C—O1C—C1C—O2C	56.97 (19)
C7A—C8A—C9A—C10A	−54.66 (18)	C5C—O1C—C1C—C2C	−61.39 (19)
Na1A^ii^—O11A—C10A—C9A	−75.4 (2)	Na1C—O3C—C2C—C3C	77.26 (15)
Na1A^ii^—O11A—C10A—C11A	45.9 (3)	Na1C—O3C—C2C—C1C	−43.25 (16)
O10A—C9A—C10A—O11A	−66.95 (18)	O2C—C1C—C2C—O3C	57.24 (18)
C8A—C9A—C10A—O11A	173.19 (14)	O1C—C1C—C2C—O3C	178.24 (14)
O10A—C9A—C10A—C11A	173.98 (14)	O2C—C1C—C2C—C3C	−63.71 (18)
C8A—C9A—C10A—C11A	54.12 (18)	O1C—C1C—C2C—C3C	57.28 (18)
C7A—O7A—C11A—C12A	−177.39 (14)	O3C—C2C—C3C—O4C	66.14 (18)
C7A—O7A—C11A—C10A	59.09 (18)	C1C—C2C—C3C—O4C	−174.54 (14)
O11A—C10A—C11A—O7A	−175.80 (13)	O3C—C2C—C3C—C4C	−173.68 (14)
C9A—C10A—C11A—O7A	−54.94 (17)	C1C—C2C—C3C—C4C	−54.35 (18)
O11A—C10A—C11A—C12A	63.94 (18)	Na1C^vi^—O5C—C4C—C3C	−82.3 (2)
C9A—C10A—C11A—C12A	−175.20 (14)	Na1C^vi^—O5C—C4C—C5C	39.3 (3)
O7A—C11A—C12A—O12A	−67.61 (19)	O4C—C3C—C4C—O5C	−67.22 (18)
C10A—C11A—C12A—O12A	53.6 (2)	C2C—C3C—C4C—O5C	172.33 (14)
O5B^iii^—Na1B—O2B—C1B	99.63 (14)	O4C—C3C—C4C—C5C	173.99 (14)
O9B—Na1B—O2B—C1B	−120.26 (12)	C2C—C3C—C4C—C5C	53.55 (18)
O3B—Na1B—O2B—C1B	13.02 (11)	C1C—O1C—C5C—C6C	−177.14 (15)
O11B^iv^—Na1B—O2B—C1B	160.56 (12)	C1C—O1C—C5C—C4C	59.47 (18)
O8B—Na1B—O2B—C1B	−53.49 (14)	O5C—C4C—C5C—O1C	−175.59 (14)
O5B^iii^—Na1B—O3B—C2B	−113.75 (12)	C3C—C4C—C5C—O1C	−54.45 (18)
O9B—Na1B—O3B—C2B	93.85 (13)	O5C—C4C—C5C—C6C	63.99 (19)
O2B—Na1B—O3B—C2B	19.76 (12)	C3C—C4C—C5C—C6C	−174.86 (15)
O11B^iv^—Na1B—O3B—C2B	−34.83 (17)	O1C—C5C—C6C—O6C	−65.9 (2)
O8B—Na1B—O3B—C2B	150.40 (12)	C4C—C5C—C6C—O6C	55.1 (2)
O5B^iii^—Na1B—O8B—C7B	141.71 (11)	Na1C—O8C—C7C—O7C	−163.83 (10)
O9B—Na1B—O8B—C7B	21.16 (11)	Na1C—O8C—C7C—C8C	−43.68 (15)
O3B—Na1B—O8B—C7B	−116.54 (11)	C11C—O7C—C7C—O8C	57.97 (18)
O2B—Na1B—O8B—C7B	−57.15 (13)	C11C—O7C—C7C—C8C	−61.16 (18)
O11B^iv^—Na1B—O8B—C7B	68.46 (14)	Na1C—O9C—C8C—C7C	−41.39 (16)
O5B^iii^—Na1B—O9B—C8B	−75.30 (13)	Na1C—O9C—C8C—C9C	79.42 (15)
O3B—Na1B—O9B—C8B	72.82 (14)	O8C—C7C—C8C—O9C	55.19 (17)
O2B—Na1B—O9B—C8B	136.78 (12)	O7C—C7C—C8C—O9C	177.13 (13)
O11B^iv^—Na1B—O9B—C8B	−138.59 (12)	O8C—C7C—C8C—C9C	−65.12 (17)
O8B—Na1B—O9B—C8B	10.55 (11)	O7C—C7C—C8C—C9C	56.82 (18)
Na1B—O2B—C1B—O1B	−160.91 (10)	O9C—C8C—C9C—O10C	67.30 (17)
Na1B—O2B—C1B—C2B	−40.78 (16)	C7C—C8C—C9C—O10C	−173.15 (13)
C5B—O1B—C1B—O2B	57.95 (19)	O9C—C8C—C9C—C10C	−174.30 (14)
C5B—O1B—C1B—C2B	−60.91 (18)	C7C—C8C—C9C—C10C	−54.74 (18)
Na1B—O3B—C2B—C1B	−47.04 (16)	Na1C^v^—O11C—C10C—C9C	−17.0 (3)
Na1B—O3B—C2B—C3B	73.84 (15)	Na1C^v^—O11C—C10C—C11C	104.0 (2)
O2B—C1B—C2B—O3B	56.69 (18)	O10C—C9C—C10C—O11C	−66.05 (18)
O1B—C1B—C2B—O3B	178.18 (14)	C8C—C9C—C10C—O11C	174.44 (14)
O2B—C1B—C2B—C3B	−64.71 (18)	O10C—C9C—C10C—C11C	174.07 (14)
O1B—C1B—C2B—C3B	56.78 (18)	C8C—C9C—C10C—C11C	54.57 (18)
O3B—C2B—C3B—O4B	65.90 (18)	C7C—O7C—C11C—C12C	−176.51 (14)
C1B—C2B—C3B—O4B	−175.12 (14)	C7C—O7C—C11C—C10C	61.14 (18)
O3B—C2B—C3B—C4B	−173.34 (14)	O11C—C10C—C11C—O7C	−177.64 (13)
C1B—C2B—C3B—C4B	−54.36 (18)	C9C—C10C—C11C—O7C	−56.78 (17)
Na1B^iv^—O5B—C4B—C3B	−76.5 (2)	O11C—C10C—C11C—C12C	62.72 (18)
Na1B^iv^—O5B—C4B—C5B	45.2 (3)	C9C—C10C—C11C—C12C	−176.41 (14)
O4B—C3B—C4B—O5B	−66.17 (18)	O7C—C11C—C12C—O12C	−68.07 (19)
C2B—C3B—C4B—O5B	173.62 (13)	C10C—C11C—C12C—O12C	52.5 (2)

Symmetry codes: (i) −*y*+1, *x*−*y*+1, *z*+1/3; (ii) −*x*+*y*, −*x*+1, *z*−1/3; (iii) −*y*+2, *x*−*y*+1, *z*+1/3; (iv) −*x*+*y*+1, −*x*+2, *z*−1/3; (v) −*x*+*y*+1, −*x*+1, *z*−1/3; (vi) −*y*+1, *x*−*y*, *z*+1/3.

### Hydrogen-bond geometry (Å, °)

**Table:**

*D*—H···*A*	*D*—H	H···*A*	*D*···*A*	*D*—H···*A*
O2A—H2A···O12B^vii^	0.85	1.79	2.630 (3)	172
O2B—H2B···O12C^viii^	0.85	1.79	2.640 (3)	174
O2C—H2C···O6A^ix^	0.85	1.82	2.665 (3)	176
O3A—H3A···O1B	0.85	1.91	2.753 (2)	169
O3B—H3B···O1C^vi^	0.85	1.90	2.727 (2)	163
O3C—H3C···O7A^x^	0.85	1.94	2.755 (2)	161
O4A—H4A···Cl2^xi^	0.82	2.34	3.158 (2)	174
O4B—H4B···Cl3^xii^	0.82	2.45	3.270 (2)	173
O4C—H4C···Cl1	0.82	2.47	3.256 (2)	161
O5A—H5A···Cl2^xiii^	0.83	2.35	3.151 (1)	162
O5B—H5B···O10B^ix^	0.85	1.98	2.828 (2)	175
O5C—H5C···O10C^xiv^	0.85	1.94	2.773 (2)	165
O6A—H6A···Cl2^xiii^	0.82	2.33	3.144 (2)	174
O6B—H6B···Cl3^ii^	0.82	2.41	3.220 (2)	170
O6C—H6C···O8B^v^	0.82	2.33	2.696 (3)	108
O8A—H8A···O6B	0.85	1.88	2.726 (3)	177
O8B—H8B···O6C^vi^	0.93	2.10	2.696 (3)	121
O8C—H8C···O12A^x^	0.85	1.85	2.699 (2)	173
O9A—H9A···O7B^vii^	0.85	1.97	2.786 (3)	162
O9B—H9B···O7C^viii^	0.85	1.97	2.798 (2)	166
O9C—H9C···O1A^ix^	0.85	1.94	2.775 (2)	168
O10A—H10A···Cl2^i^	0.82	2.51	3.245 (2)	150
O10B—H10B···Cl3^xv^	0.82	2.33	3.147 (2)	171
O10C—H10C···Cl1^v^	0.82	2.48	3.144 (2)	139
O11A—H11A···O4A^vii^	0.85	2.03	2.819 (3)	155
O11B—H11B···Cl3^xi^	0.81	2.43	3.159 (2)	150
O11C—H11C···Cl1^xvi^	0.83	2.36	3.166 (2)	164
O12A—H12A···Cl2	0.82	2.45	3.229 (2)	159
O12B—H12B···Cl3^xi^	0.82	2.37	3.135 (2)	156
O12C—H12C···Cl1^xvi^	0.82	2.32	3.138 (2)	173

Symmetry codes: (vii) −*y*+1, *x*−*y*+1, *z*−2/3; (viii) *x*, *y*+1, *z*+1; (ix) −*y*+2, *x*−*y*+1, *z*−2/3; (vi) −*y*+1, *x*−*y*, *z*+1/3; (x) *x*, *y*−1, *z*; (xi) −*x*+*y*, −*x*+1, *z*+2/3; (xii) *x*+1, *y*, *z*; (xiii) *x*, *y*, *z*+1; (xiv) −*x*+*y*+1, −*x*+1, *z*+2/3; (ii) −*x*+*y*, −*x*+1, *z*−1/3; (v) −*x*+*y*+1, −*x*+1, *z*−1/3; (i) −*y*+1, *x*−*y*+1, *z*+1/3; (xv) −*y*+2, *x*−*y*+2, *z*+1/3; (xvi) −*y*+1, *x*−*y*, *z*−2/3.
